# Comparison of virulence gene profiles and genomic fingerprints of *Vibrio cholerae* O1 and non-O1/non-O139 isolates from diarrheal patients in southern Thailand

**DOI:** 10.1186/s41182-018-0113-x

**Published:** 2018-09-05

**Authors:** Sakrapee Tulatorn, Sutima Preeprem, Varaporn Vuddhakul, Pimonsri Mittraparp-arthorn

**Affiliations:** Department of Microbiology, Faculty of Science, Prince of Songkla University, 15 Kanjanavanich Rd., Hat Yai, Songkhla, 90110 Thailand

**Keywords:** Molecular typing, Multi-locus variable-number of tandem-repeat analysis, Pulsed-field gel electrophoresis, *Vibrio cholerae*, Virulence gene

## Abstract

**Background:**

*Vibrio cholerae* is associated with severe watery diarrheal disease among people in many parts of the world, including the coastal provinces of Southern Thailand. There are relatively few studies focusing on the genetic characterization among *V. cholerae* isolates in this region. Therefore, this study aimed at exploring the presence of virulence genes and DNA fingerprints among *V. cholerae* O1 and non**-**O1/non**-**O139 isolates obtained from clinical samples in four southern coastal provinces during the period of 2001**–**2009 (*n* = 21).

**Results:**

All *V. cholerae* O1 isolates possessed *ctxA*, *tcpA*, *zot*, *ace*, *hlyA*, and *vasH* genes. However, only *hlyA*, *vcsV2*, and *vasH* genes were detected in the majority of the non**-**O1/non**-**O139 isolates. All O1 isolates showed indistinguishable PCR fingerprints by arbitrarily primed (AP)**-**PCR and enterobacterial repetitive intergenic consensus (ERIC)**-**PCR regardless of the geographical area and period of isolation. However, the multi-locus variable-number of tandem-repeat analysis (MLVA) could differentiate these O1 isolates (*n* = 11) into eight profiles. Isolates exhibiting an undistinguished MLVA profile also showed identical pulsed-field gel electrophoresis (PFGE). In addition, the O1 isolates were grouped into the same cluster by all methods used in this study.

**Conclusions:**

This study demonstrated the presence of virulence genes and genetic diversity among different serogroups of *V. cholerae* isolates from clinical samples in southern Thailand. *V. cholerae* O1 isolated over a period of multiple years were genetically related, suggesting that they had a clonal origin, whereas non**-**O1/non**-**O139 isolates could have evolved independently.

## Background

*Vibrio cholerae* is one of the endemic pathogens causing acute diarrheal disease in several parts of the world. During the past 10 years, the highest documented outbreaks in Thailand occurred in 2001, 2004, 2007, 2010, and 2016 [1], and approximately 31% of reported cholera cases in 2007 occurred in the southern regions [2]. Many of the reported cholera cases originated from the southern coastal provinces which are located on the Malay Peninsula. The *V. cholerae* infections in the southern region are almost exclusively caused by consumption of contaminated seafood or poor sanitation and hygiene among fishing communities and alien laborers.

Throughout history, *V. cholerae* O1 serogroup has dominated the affected geographical regions. Toxigenic *V. cholerae* serogroups O1 and O139 have been reported to harbor the *ctxA* and *tcpA* genes, encoding the A subunit of cholera toxin (CT) and the major subunit of a toxin coregulated pilus (Tcp), respectively. Furthermore, the *zot*, encoding the zonula occludens toxin (Zot), and *ace* encoding the accessory cholera enterotoxin (Ace) were also reported to be involved in the pathogenesis of O1 and O139 serogroups. However, these genes were reported to be absent in the non-O1/non-O139 isolates [3, 4]. The *stn/sto* and *hlyA* genes, encoding a non-O1 heat-stable enterotoxin (NAG-ST) and the El Tor-like hemolysin, respectively, have also been reported to associate with non-O1/non-O139 infections [5]. In addition, the type III (T3SS) and type VI (T6SS) secretion systems are considered as additional important virulence factors of *V. cholerae* [6, 7].

Among the methods for studying the relatedness or differentiations among the *V. cholerae* isolates, pulsed-field gel electrophoresis (PFGE) is considered to be the gold standard [8–10]. Unfortunately, this method is time-consuming and requires specialized equipment. Polymerase chain reaction (PCR)-based typing methods, in comparison to PFGE, are easier, faster, and less expensive. These methods, including box elements PCR (BOX-PCR), arbitrarily primed PCR (AP-PCR) [3, 4, 11], enterobacterial repetitive intergenic consensus sequence PCR (ERIC-PCR) [3, 12–14], *V. cholerae* repeats-PCR (VCR-PCR) [15, 16], and multi-locus variable-number of tandem-repeat (VNTR) analysis (MLVA) [17–20] have been evaluated for typing of *V. cholerae* isolates obtained from various geographic regions.

Southern coastal provinces of Thailand are considered to be the endemic regions for cholera. In this study, we examined *V. cholerae* isolates obtained from diarrheal patients in these areas for their virulence gene profiles and also elucidate their genetic relationships using well established PCR-based fingerprinting methods. Moreover, the discriminatory ability of each method was also determined.

## Methods

### Bacterial strain

A total of 21 *V. cholerae* isolates (VC1-VC21) from clinical samples in four southern coastal provinces were included in this study (Table 1). All isolates were obtained between 2001 and 2009 from sporadic cholera cases as part of the routine microbiological diagnosis, and were kindly provided by Hat Yai Hospital, Songkhla, Thailand. These isolates were confirmed as *V. cholerae* by *ompW*-based PCR [21]. Serogrouping was performed by slide agglutination using O1 and O139 antisera. Bacterial genomic DNA was extracted using a Genomic DNA extraction kit (Geneaid, Taiwan).

**Table 1:** Details of *V. cholerae* isolates used and their molecular characteristics

Isolate	Serogroup	Year of isolation	Location	Virulence gene profile^b^	Designated fingerprint type				VNTR allele		
					AP-PCR	ERIC-PCR	VCR-PCR	MLVA	VC0436-7	VC1650	VCA0171
VC1	O1	2001	Songkhla	A	1	1	1	1	9	13	21
VC2	O1	2001	Songkhla	A	1	1	1	1	9	13	21
VC3	O1	2001	Nakhon Si Thammarat	A	1	1	1	1	9	13	21
VC4	O1	2001	Phuket	A	1	1	1	1	9	13	21
VC5	O1	2007	Songkhla	A	1	1	2	2	10	13	22
VC6	O1	2007	Songkhla	A	1	1	2	3	9	13	22
VC7	O1	2007	Songkhla	A	1	1	2	4	9	9	21
VC8	O1	2007	Pattani	A	1	1	2	5	10	10	22
VC9	O1	2007	Pattani	A	1	1	2	6	11	11	23
VC10	O1	2009	Songkhla	A	1	1	2	7	8	9	22
VC11	O1	2009	Songkhla	A	1	1	2	8	9	9	23
VC12	NAG^a^	2001	Songkhla	B	2	2	×^c^	9	9	11	17
VC13	NAG	2001	Phuket	C	3	3	3	10	6	6	19
VC14	NAG	2007	Songkhla	E	4	4	4	11	12	3	7
VC15	NAG	2007	Songkhla	F	5	5	×	12	6	8	24
VC16	NAG	2008	Songkhla	E	6	1	5	13	7	6	13
VC17	NAG	2008	Songkhla	E	7	1	6	14	5	12	11
VC18	NAG	2009	Songkhla	E	8	1	7	15	5	5	15
VC19	NAG	2009	Songkhla	D	9	1	8	16	4	6	15
VC20	NAG	2009	Songkhla	D	9	1	8	17	5	9	16
VC21	NAG	2009	Songkhla	E	10	1	9	18	5	6	16

^a^*NAG* non-agglutinating *V. cholerae* serogroup non-O1/non-O139

^b^Virulence gene profiles are provided for direct comparison

^c^×, no DNA pattern was observed on gel electrophoresis

### PCR detection of virulence genes

*V. cholerae* isolates were screened for the presence of eight virulence genes, including *ctxA*, *tcpA*, *zot*, *ace*, *stn/sto*, *hlyA*, *vcsV2* (T3SS), and *vasH* (T6SS) genes. All primers, amplicon sizes, and PCR conditions are listed in Table 2. PCR products were visualized after electrophoresis in 1.8% agarose gel.

**Table 2:** PCR primers used in this study

Target genes	Protein product	Sequence (5′–3′)	Product size (bp)^a^	Reference
*ctxA*	Cholera toxin	CGGGCAGATTCTAGACCTCCTG CGATGATCTTGGAGCATTCCCAC	564	[45, 46]
*tcpA*	Toxin-coregulated pili	CACGATAAGAAAACCGGTCAAGAG CGAAAGCACCTTCTTTCACACGTTG TTACCAAATGCAACGCCGAATG	453 (ET) 620 (C)	[28, 46]
*zot*	Zonula occludens toxin	TCGCTTAACGATGGCGCGTTTT AACCCCGTTTCACTTCTACCCA	947	[28, 46]
*ace*	Accessory cholera enterotoxin	TAAGGATGTGCTTATGATGGACACCC CGTGATGAATAAAGATACTCATAGG	316	[47]
*stn/sto*	Heat-stable enterotoxin	TCGCATTTAGCCAAACAGTAGAAA GCTGGATTGCAACATATTTCGC	172	[28]
*hlyA*	El Tor Hemolysin	GGCAAACAGCGAAACAAATACC GAGCCGGCATTCATCTGAAT CTCAGCGGGCTAATACGGTTTA	481 (ET) 738/727 (ET/C)	[28]
*vcsV2*	ATPase (T3SS)	ATGCAGATCTTTTGGCTCACTTGATGGG ATGCGTCGACGCCACATCATTGCTTGCT	742	[6]
*vasH*	Transcriptional regulator (T6SS)	TGTTGATGGGCGAGAGTCAC ACGTGTGTGGCAGATACCAG	631	[31]

^a^*C* Classical, *ET* El Tor

### Molecular typing by AP-PCR, ERIC-PCR, and VCR-PCR

DNA from pure cultures was extracted using the Genomic DNA Mini Kit (Geneaid, Taiwan) according to the manufacturer’s instructions. AP-PCR was performed using a single nucleotide primer, Primer 2 (5′-GTTTCGCTCC) [22]. ERIC-PCR was performed as previously described using the primer pair ERIC1R (5′- ATGTAAGCTCCTGGGGATTCAC-3′) and ERIC2 (5′-AAGTAAGTGACTGGGGTGAGCG-3′) [14]. For AP-PCR and ERIC-PCR, *ExTaq* DNA polymerase (TaKaRa, Japan) was used instead of conventional *Taq* DNA polymerase. For VCR-PCR, the primers VCR-5′ (5′-TCCCTCTTGAGGCGTTTGTTAC-3′) and VCR-3′ (5′-AGCCCCTTAGGCGGGCGTTAA-3′) were used [16], and the amplification was carried out as described by Teh et al. [15]. The amplification products were analyzed by electrophoresis using a 1.5% agarose gel. DNA patterns were compared by the unweighted pair group method with arithmetic mean (UPGMA) using the Dice coefficient. A dendrogram was constructed using Bionumeric software v.7.6 (Applied Maths, Belgium).

### MLVA and data analysis

MLVA was conducted using primers and protocols described previously [17]. Three selected loci (VC0436-7, VC1650, and VCA0171) were amplified by PCR using 5′ fluorescent-labeled forward primers and analyzed by capillary electrophoresis using an ABI 3730xl Genetic Analyzer (Applied BioSystems, USA). Fragment sizes from each VNTR locus were determined using the GeneMapper software (v.4.1) (Applied BioSystems, USA) and were converted into copy numbers using the following equation: Number of repeats (bp) = [fragment size (bp) − flanking regions (bp)]/repeat size (bp) [23]. The repeat copy numbers were analyzed, and a dendrogram was constructed by Bionumeric software v.7.6 (Applied Maths, Belgium).

### PFGE

Isolates with undistinguished DNA profile by the above methods were further determined by PFGE using the PulseNet standardized protocol for *V. cholerae* [9]. Chromosomal DNA of *V. cholerae* was digested with restriction enzyme *Not* I (NEB, USA), and the digested DNA fragments were separated on 1% Pulse-Field Certified agarose gel by CHEF-DRIII system (Bio-Rad Laboratories, California, USA). The gel was stained with ethidium bromide, and the DNA patterns were analyzed using Bionumeric software v.7.6 (Applied Maths, Belgium).

### Discriminatory index and typeability

The discriminatory powers were calculated using previously published formula [24]. The polymorphism information index or Nei’s diversity index (DI) of each VNTR locus was calculated individually using the formula: *D* = 1 − Σ (allele frequency)^2^ [25]. Typeability was calculated from the formula recommended previously [26].

## Results

### Virulence gene profiles

A total of six virulence gene profiles (A to F) were observed (Table 3). The most prevalent virulence genes detected were *hlyA* (95%) and *vasH* (95%), and the distribution of virulence genes was related to serogroups. All of the O1 isolates were positive for the *ctxA*, *tcpA*, *zot*, *ace*, *hlyA*, and *vasH* virulence genes. None of the O1 isolates were positive for the *stn/sto* and *vcsV2* (T3SS) genes. All non-O1/non-O139 isolates were negative for *tcpA* gene. Interestingly, *ctxA*, *zot*, and/or *ace* genes were detected in two isolates of *V. cholerae* non-O1/non-O139 (VC12 and VC13). In this study, one non-O1/non-O139 isolate (VC15) was negative for all virulence gene tested (Table 3).

**Table 3:** Distribution of virulence-associated genes among *V. cholerae* isolates

Virulence gene profile (*n*)	Presence of genes^a^								Serogroup
	*ctxA*	*tcpA*	*zot*	*ace*	*stn/sto*	*hlyA*	*vcsV2*	*vasH*	
A (11)	+	+	+	+	–	+	–	+	O1
B (1)	+	–	+	+	–	+	–	+	NAG^b^
C (1)	+	–	+	–	–	+	+	+	NAG
D (2)	–	–	–	–	+	+	+	+	NAG
E (5)	–	–	–	–	–	+	+	+	NAG
F (1)	–	–	–	–	–	–	–	–	NAG
% Prevalence	62	52	62	57	10	95	38	95	

^a^+, present; −, absent

^b^*NAG* non-agglutinating *V. cholerae* serogroup non-O1/non-O139

### Molecular typing of *V. cholerae* isolates by AP-PCR, ERIC-PCR, and VCR-PCR

**Fig. 1:**
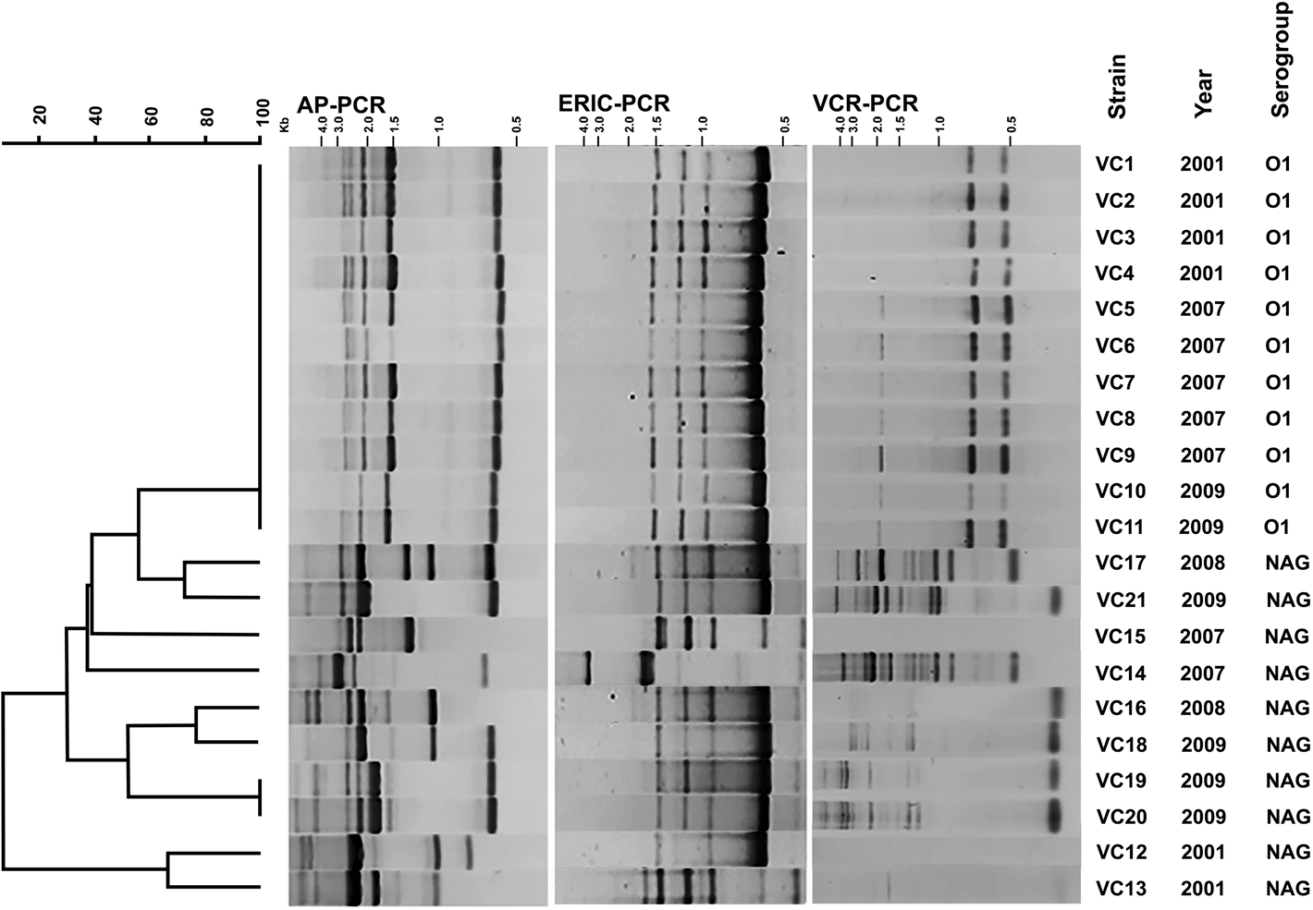
Dendrogram and PCR fingerprints for comparison of *V. cholerae* isolates. The dendrogram is based on the PCR fingerprints generated by AP-PCR. The PCR fingerprints of ERIC-PCR and VCR-PCR are provided for direct comparison

The 21 *V. cholerae* isolates were differentiated into 10, 5, and 9 profiles based on DNA patterns obtained by AP-PCR, ERIC-PCR, and VCR-PCR, respectively (Table 1). Good correlation was observed between the fingerprinting results of AP-PCR, ERIC-PCR, and VCR-PCR. All of the O1 isolates (*n* = 11) yielded identical fingerprints by AP-PCR and ERIC-PCR, and highly similar patterns by VCR-PCR (Fig. 1). In addition, only AP-PCR and VCR-PCR could distinguish serogroup O1 from non-O1/non-O139 isolates. The fingerprints of non-O1/non-O139 isolates tended to be more diverse, but were not correlated with geographical areas and period of isolations. In this study, AP-PCR and VCR-PCR of non-O1/non-O139 isolates resulted in amplification of more DNA fragments than ERIC-PCR, although no VCR-PCR product was observed from two isolates (VC12 and VC15). The dendrograms generated for all *V. cholerae* isolates based on AP-PCR method are shown in Fig. 1. These methods showed low discriminatory power in typing, especially among the O1 isolates.

### MLVA and phylogenetic analysis

All *V. cholerae* isolates were distinguished into 18 MLVA profiles (Table 1). All profiles were represented by a single isolate except for MLVA type 1 that was represented by four isolates (VC1, VC2, VC3, and VC4). The PFGE profiles of these four isolates showed identical restriction patterns (data not shown). The copy numbers of VNTR alleles in VC0436-7, VC1650, and VCA0171 loci ranged from 4–12, 3–13, and 7–24 repeats, respectively (Table 1). In total, 9, 9, and 11 alleles were observed in VC0436-7, VC1650, and VCA0171, respectively.

**Fig. 2:**
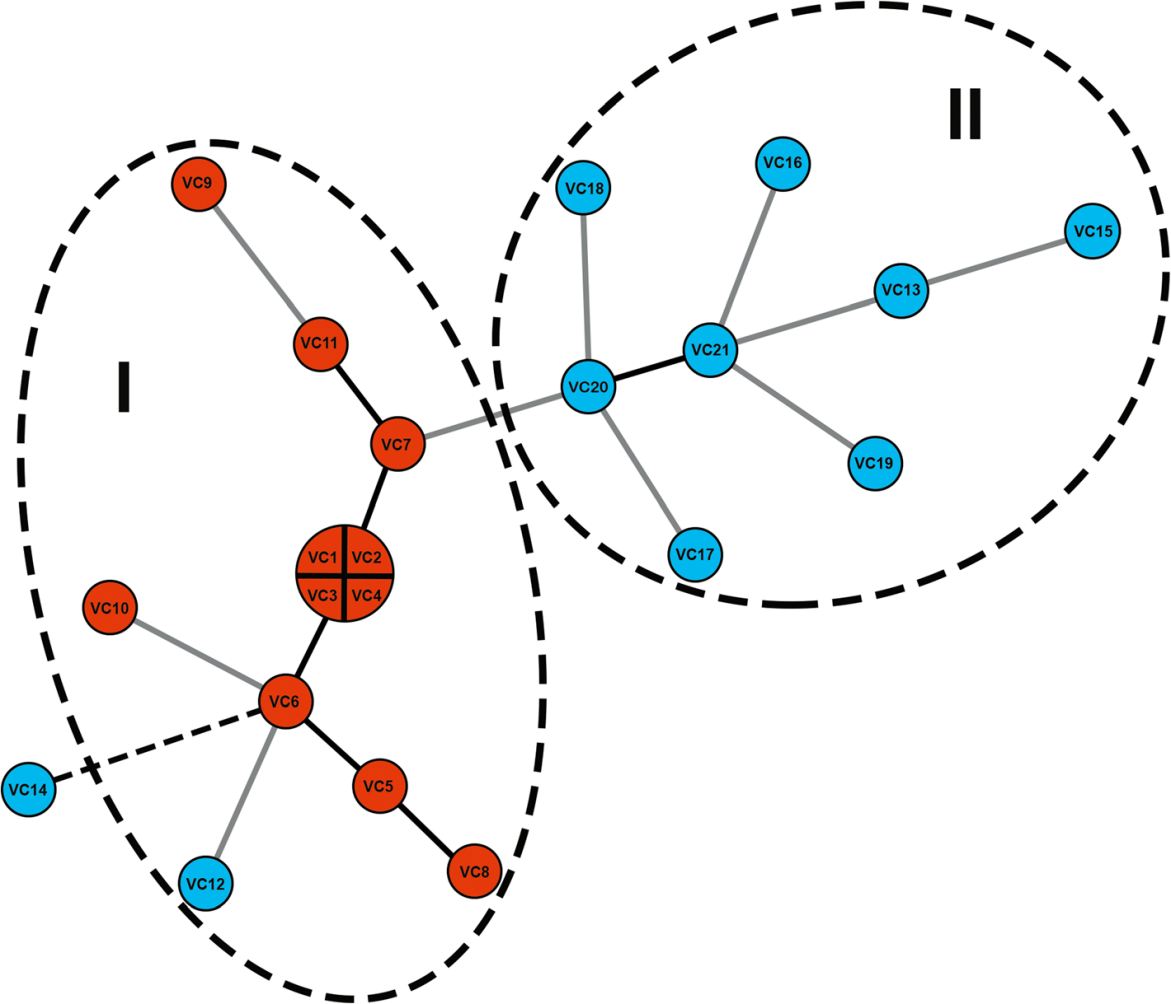
Minimum spanning tree of the 21 *V. cholerae* isolates based on MLVA profiles. Each circle represents a unique MLVA profile. The color of the circles corresponds to serogroups. The circle size is proportional to the number of isolates. A distance of one locus between two MLVA profiles is indicated by a thick line, a distance of two loci is indicated by a gray line, and a distance of three loci is indicated by a dot line. The dotted circle indicates the MLVA clusters (I and II)

To demonstrate the relationships among *V. cholerae* isolates, a minimum spanning tree (MST) was constructed based on the MLVA profiles (Fig. 2). All O1 isolates were grouped into the same cluster (cluster I). In this cluster, four of the O1 isolates (VC1, VC2, VC3, and VC4) obtained during 2001 from different regions (Songkhla, Nakhon Si Thammarat, and Phuket) shared the same profile (9-13-21). Interestingly, a non-O1/non-O139 isolate (VC12) obtained during the same period was in this cluster. Cluster II composed of non-O1/non-O139 isolates which were genetically diverse (Fig. 2).

### Discriminatory index and typeability

In this study, the comparison of Simpson’s index of diversity revealed that MLVA exhibited the highest discrimination value (0.97) (Table 4). VCA0171 was identified to be the most polymorphic loci with a much higher DI (0.89) than the others. VC0436-7 and VC1650 had the DI value of 0.79 and 0.83, respectively. All typing methods, except VCR-PCR, were able to type 100% of the isolates examined (Table 4).

**Table 4:** Discriminatory power and typeability of fingerprinting methods used in this study for typing of *V. cholerae* isolates

Method	Discriminatory power		Typeability (%)
	No. of type	DI^a^	
AP-PCR	10	0.73	100
ERIC-PCR	5	0.35	100
VCR-PCR	9	0.86	90
MLVA	18	0.97	100

^a^Simpson’s diversity index

## Discussion

In this study, *V. cholerae* O1 and non-O1/non-O139 obtained from clinical samples in southern Thailand were molecularly characterized to obtain an understanding of their virulence and genetic relationship. The distribution of *ctxA*, *tcpA*, *zot*, and *ace* genes was strongly correlated with O1 serogroup. Although these virulence genes are rarely detected from the non-O1/non-O139 *V. cholerae* isolates, *ctxA*, *zot*, and/or *ace* genes were detected in two of the non-O1/non-O139 isolates (VC12 and VC13) examined in this study. The occurrence of *ctxA*-positive non-O1/non-O139 isolates was found in India and Brazil [27, 28].

In this study, *vcsV2* (T3SS) was not detected in all clinical isolates of *V. cholerae* O1. This supports the previous studies from Rahman et al. (2008) which demonstrated that most clinical isolates of *V. cholerae* O1 and O139 were negative for T3SS [29]. The presence of T3SS in majority of non-O1/non-O139 isolates was also reported in Bangladesh, China, Nigeria, Germany, and Austria [29–32]. However, the incidence of T3SS among *V. cholerae* non-O1/non-O139 isolated in this study was 73% (8/11) which is higher than that reported in India (31.5%) [6] and Bangladesh (38.9%) [33]. The role of T3SS in human infections by non-O1/non-O139 was reported to correlate with increased hemolytic titers and motility [6]. Thus, the presence of T3SS might be essential not only for environmental fitness but also for the pathogenesis of non-O1/non-O139 in human hosts. In this study, *vasH* (T6SS) gene was detected in almost all isolates, regardless of serogroups. This supports the previous report which demonstrated that T6SS are conserved between *V. cholerae* O1, O139, and non-O1/non-O139 [34]. However, T6SS expression differs between strains and is strictly regulated in O1 and O139 serogroups. The role of T6SS was reported to play a role in *V. cholerae* fitness in the aquatic environments, and it is repressed at initial infection in human hosts [34].

Several established DNA banding pattern-based genotyping methods, including AP-PCR, ERIC-PCR, and VCR-PCR, were used to analyze the genetic relatedness of *V. cholerae* O1 and non-O1/non-O139 isolates. The O1 isolates (VC1-VC11) in this study yield DNA fingerprints that were identical by AP-PCR or highly similar by VCR-PCR but distinct from those of non-O1/non-O139 isolates (VC12-VC21). However, ERIC-PCR could not differentiate some of non-O1/non-O139 (VC16-VC21) from O1 isolates. Low discrimination of these methods in *V. cholerae* O1 typing was also reported among the strains isolated from India, Malaysia, Taiwan, and Iran [11, 35–37]. This may be due to the highly conserved genomes among the *V. cholerae* O1 serogroup. Thus, these results confirmed the clonality among isolates of *V. cholerae* O1.

The MLVA method was shown to be more effective for studying the genetic relationships among *V. cholerae* isolates than other PCR-based typing methods [38] because this typing method relies on the detection of multiple tandem repeats on bacterium genomes that evolve rapidly [17]. To increase the accuracy of this method, capillary DNA sequencing of PCR fragments from each VNTR locus is used instead of agarose gel electrophoresis. In previous reports, the VNTR locus VCA0171 (located on chromosome II) of *V. choleare* Indian and Haiti isolates had the greatest diversity compared with VC0436-7 and VC1650 loci [18, 39]. The discriminatory ability of VcA VNTR based on VCA0171 locus for typing of *V. cholerae* strains was better than PFGE [40]. The results from this study support these findings.

In this study, MLVA showed an agreement with epidemiological data. Four O1 isolates that shared the same PFGE pattern and MLVA profile (VC1, VC2, VC3, and VC4) were obtained during the same period. The association between *V. cholerae* serogroups, year and site of isolation, and the genotypic clusters was previously reported by other studies [18, 19]. In this study, it is possible that these areas were probably contaminated with the same clone of *V. cholerae* and this strain may be responsible for the occurrence of cholera in the study regions during that period.

An isolate of non-O1/non-O139 (VC12) was grouped together with O1 isolates in MLVA cluster I. This isolate harbored all O1-specific virulence genes, except *tcpA* which may be due to the variation in the primer binding site. This isolate may has evolved from O1 serogroup strain by O-antigen shifting during the evolutionary step as previously occurred in *V. cholerae* serogroups O139, O26, and O37 [41–43]. It is possible that new toxigenic non-O1/non-O139 *V. cholerae* isolate with epidemic potential may emerge in the future. In contrast to VC12, the isolate VC13 possessed non-O1/non-O139 background (MLVA cluster II) (Fig. 2). This isolate may acquire the O1-specific virulence genes, *ctxA* and/or *zot*, by horizontal gene transfer. The presence of *ctxA* and/or *zot* genes among non-O1/non-O139 isolates demonstrated the potential of natural genetic exchange in this organism which may have occurred in aquatic ecosystems or inside the human host [27, 44]. Interestingly, the repeat numbers of VC0436-7 locus in one of the non-O1/non-O139 isolates (VC14) were related to those of the O1 isolates. Thus, isolate VC14 was excluded from cluster II. Clusters may change if more VNTR loci were included in the analysis. Further study is needed to support the relationship among these isolates.

## Conclusion

The genetic relationship within serogroups of clinical *V. cholerae* isolates was demonstrated in this study. However, most of the non-O1/non-O139 isolates were very heterogeneous regarding their virulence gene patterns and DNA fingerprints. The fingerprinting methods should be applied according to their serogroups and sources of isolation. AP-PCR may be enough for typing among non-O1/non-O139 isolates. However, MLVA which exhibited higher discriminatory power than those methods should be used for typing of *V. cholerae* serogroup O1. Understanding the association between bacterial virulence characteristics, genotypes, year, and source of isolation is necessary for epidemiological surveillance of *V. cholerae* infections.

## Abbreviations

Ace: Accessory cholera enterotoxin
AP-PCR: Arbitrarily primed-PCR
CT: Cholera toxin
DI: Diversity index
ERIC-PCR: Enterobacterial repetitive intergenic consensus-PCR
MLVA: Multi-locus variable-number of tandem-repeat analysis
NAG: Non-agglutinating *V. cholerae* serogroup non-O1/non-O139
NAG-ST: Non-O1 heat-stable enterotoxin
PCR: Polymerase chain reaction
PFGE: Pulsed-field gel electrophoresis
T3SS: Type III secretion systems
T6SS: Type VI secretion systems
Tcp: Toxin coregulated pilus
UPGMA: Unweighted pair group method with arithmetic mean
VCR-PCR: *V. cholerae* repeats-PCR
VNTR: Variable number of tandem repeat
Zot: Zonula occludens toxin
